# Inulin Reverses Intestinal Mrp2 Downregulation in a Diet-Induced Obesity Mouse Model: Role of Intestinal Microbiota as a Pivotal Modulator

**DOI:** 10.3390/pharmaceutics17121575

**Published:** 2025-12-06

**Authors:** Felipe Zecchinati, Laura Ricardi, Víctor Blancato, Emmanuel Pereyra, Maite Arana, Carolina Ghanem, Virginia Perdomo, Silvina Villanueva

**Affiliations:** 1 Instituto de Fisiología Experimental (IFISE-CONICET), Facultad de Ciencias Bioquímicas y Farmacéuticas, Universidad Nacional de Rosario, Rosario 2000, Argentina; fz_venado@hotmail.com (F.Z.); emmanuelisidorop@gmail.com (E.P.); arana@ifise-conicet.gov.ar (M.A.); 2 Laboratorio de Fisiología y Genética de Bacterias Lácticas, Instituto de Biología Molecular y Celular de Rosario (IBR-CONICET), Facultad de Ciencias Bioquímicas y Farmacéuticas, Universidad Nacional de Rosario, Rosario 2000, Argentina; blancato@ibr-conicet.gov.ar; 3 Laboratorio de Biotecnología e Inocuidad de los Alimentos, Facultad de Ciencias Bioquímicas y Farmacéuticas-Municipalidad de Granadero Baigorria, Universidad Nacional de Rosario, Rosario 2000, Argentina; 4 Instituto de Investigaciones Farmacológicas (ININFA-CONICET), Facultad de Farmacia y Bioquímica, Universidad de Buenos Aires, Buenos Aires C1113AAD, Argentina; cghanem@ffyb.uba.ar; 5 Área Parasitología, Facultad de Ciencias Bioquímicas y Farmacéuticas, Universidad Nacional de Rosario, Rosario 2000, Argentina; 6 Consejo Nacional de Investigaciones Científicas y Técnicas (CONICET), Rosario 2000, Argentina

**Keywords:** intestine, obesity, microbiota, dysbiosis, Mrp2, inulin

## Abstract

**Background**: The intestinal microbiota (IM) modulates host physiology, and its alteration (dysbiosis) is associated with numerous diseases, including obesity. This condition influences the pharmacokinetics of drugs prescribed for related comorbidities, although the underlying mechanisms remain poorly understood. Mrp2, an essential ABC transporter of the intestinal biochemical barrier, regulates the absorption of dietary toxins and orally administered drugs, modulating their bioavailability. However, its regulation in the obesity context is poorly characterized, and the role of IM alteration in this process remains unknown. **Objective**: To evaluate the role of the IM as a key factor, along with downstream candidate mediators, in the regulation of Mrp2 under obesity conditions. **Methods**: Male C57BL/6 mice were fed either a control diet or High-Fat Diet (HFD) for 8 weeks, followed by 2 weeks with or without 5% inulin, a well-known prebiotic, supplementation. Metabolic and biochemical parameters were evaluated. Intestinal barrier integrity, inflammatory cytokines, oxidative stress (OS) markers, and plasma endotoxin levels were assessed. Mrp2 expression was analyzed at mRNA and protein levels, and transporter activity was determined using the everted intestinal sac model. Fecal microbiota composition was characterized by 16S rRNA sequencing. **Results**: HFD feeding induced obesity, insulin resistance, hyperglycemia, dyslipidemia, intestinal dysbiosis, elevated endotoxemia, barrier dysfunction, inflammation, and OS. These alterations were associated with a marked downregulation of Mrp2 expression and activity. Inulin supplementation restored IM composition, improved metabolic and intestinal parameters, and reduced inflammation and OS. These positive changes correlated with normalization of Mrp2. **Conclusions**: Our findings provide the first evidence that intestinal dysbiosis, inflammation, and OS act as a central regulatory axis of intestinal Mrp2 in obesity, with the IM functioning as a key modulator.

## 1. Introduction

The small intestine plays a crucial role in the digestion and absorption of nutrients from food, while simultaneously acting as a barrier against the uptake of toxins, bacterial metabolites, and orally ingested xenobiotics. This protective function is largely attributed to the ‘intestinal biochemical barrier’, in which multidrug resistance-associated protein 2 (Mp2/Abcc2) plays a pivotal role. Mrp2 is a drug efflux pump belonging to the ATP-Binding Cassette (ABC) transporter superfamily, constitutively expressed on the apical membrane of enterocytes, primarily at the villus tips of the proximal jejunum [1]. Its strategic location on the apical membrane of intestinal epithelial cells enables it to serve as a first line of defense by facilitating the excretion of potentially toxic compounds, such as environmental pollutants, and by limiting the oral bioavailability of many drugs. Thus, Mrp2 expression modulation can significantly impact the local and systemic concentrations of these compounds, thereby affecting their toxicity or efficacy and safety [2,3]. Mrp2 undergoes transcriptional and post-transcriptional modulation in response to various internal and external signals, including changes associated with particular physiological or pathological states [4].

Concomitantly, the intestine harbors the IM (intestinal microbiota), mainly composed of the phyla Firmicutes, Bacteroidetes, Actinobacteria, and Proteobacteria, with a density gradient that grows from the stomach (around 10^3^–10^4^ bacteria per gram) to the colon (about 10^12^–10^14^ bacteria per gram). The relationship between the IM and human health became one of the most important scientific discoveries of the past decade [5,6]. Increasing evidence has demonstrated that the composition and functionality of the intestinal microbial community influence the host’s health [7]. Therefore, imbalances in the composition or function of the IM (dysbiosis) has been associated with the development of several human diseases, including its role in the pathogenesis of obesity [8,9,10,11].

Obesity, a chronic metabolic disorder, represents a major public health issue worldwide, reaching epidemic proportions. Significantly, it is a risk factor for the development of metabolic syndrome (MS), diabetes, cancer, and other conditions [12,13]. Research indicates that HFD (high-fat diet), typical of Western dietary habits, is associated with both IM dysbiosis and the development and progression of obesity, referred to as “obese microbiota” [5,6]. In this regard, HFD is probably the most potent driver of IM dysbiosis, altering rapid (within 24 to 48 h) and profoundly the composition and function of microbial communities throughout the intestine. From a pathophysiological perspective, HFD-induced intestinal dysbiosis is characterized by an increased abundance of Lipopolysaccharide (LPS)-producing bacteria and a concurrent reduction in protective genera that maintain intestinal barrier integrity. These microbial community alterations are accompanied by altered levels of key short-chain fatty acids (SCFAs), which are essential for intestinal homeostasis. Collectively, these alterations trigger early intestinal events, including increased pro-inflammatory cytokine secretion, inflammation-linked oxidative stress (OS), tight junction disruption, and enhanced intestinal permeability. These disturbances facilitate the translocation of LPS into the bloodstream, promoting metabolic endotoxemia, systemic inflammation, and OS, ultimately contributing to the metabolic dysfunctions characteristic of diet-induced obesity [14,15,16]. Consistent with these observations, the interplay among HFD-induced inflammation, OS, metabolic disorders, and the IM has been suggested to be mechanistically dependent on LPS [17,18].

Interestingly, we have previously observed that the intestinal Mrp2 regulation in LPS-induced endotoxemia, involves a combination of transcriptional and post-transcriptional mechanisms, with IL-1β mediating the endocytic internalization of Mrp2 in this context [19]. Similarly, we demonstrated a significant decrease in Mrp2 expression and activity in MS-like conditions in rats fed a fructose-rich diet (FRD), with inflammation and OS acting as key mediators [20,21]. Although the contribution of dysbiosis was not specifically evaluated in either model, these findings together with existing evidence [22,23], suggest that alterations in the IM may influence Mrp2 regulation not only under those conditions but also in the current HFD model, with inflammation and OS likely operating as mediators of this regulation.

Human and animal studies have shown that obesity and HFD are associated with significant alterations in the pharmacokinetic and pharmacodynamic profiles of drugs commonly prescribed for obesity-related comorbidities [24]. Likewise, variability in drug responses has been associated with differences in IM composition [25]. Although the underlying mechanisms remain incompletely understood in both cases, it has been consistently demonstrated that the IM modulates the activity of drug-metabolizing enzymes and transporters [26,27,28].

Recent studies further support the existence of an IM–inflammation–OS axis capable of influencing Mrp2 regulation [29,30,31]. In models of metabolic and toxicant-induced liver injury, interventions that restore microbial balance and attenuate inflammation and OS have been shown to normalize efflux transporters, including Mrp2, largely through pathways involving FXR and Nrf2 [29,30,31]. While these observations are derived from hepatic models, they provide consistent mechanistic evidence supporting the existence of a comparable regulatory axis modulating Mrp2 at the intestinal level. Notably, intestinal Mrp2 remains comparatively understudied, despite its critical role in the first-pass clearance of xenobiotics, including numerous clinically relevant drugs. Furthermore, although recent evidence indicates that alterations in IM composition can influence intestinal Mrp2 expression [32], whether HFD-associated dysbiosis acts as a key upstream driver of its regulation, as well as the potential mediating role of inflammation and OS, remains to be defined. This underscores the pressing need for further investigation to elucidate the molecular mechanisms governing intestinal Mrp2 regulation under obesogenic conditions.

In this dysbiotic environment, prebiotics, such as inulin, represent a promising therapeutic approach, as they can beneficially modulate the IM and its metabolites, specifically SCFAs, improving intestinal barrier function and reducing local and systemic inflammation and metabolic dysfunctions associated with obesity [33,34].

Based on current knowledge, we hypothesize that IM dysbiosis is a key driver of intestinal Mrp2 downregulation in diet-induced obesity, impairing its barrier function via downstream factors, with inflammation and OS as the main candidates. Inulin administration, by reversing dysbiosis and restoring parameters associated with intestinal homeostasis, consequently, reestablishes the Mrp2-dependent barrier function, supporting the central role of the intestinal microbiota in its regulation. To investigate this, we initially conducted a comprehensive analysis of the Mrp2 regulation in our experimental obesity model, including mRNA and protein expression as well as functional activity, an approach not previously reported.

Unlike the study by Lu et al. (2019) [35], which described alterations in the expression of intestinal drug transporters after chronic HFD feeding without evaluating their functional consequences or the underlying molecular mechanisms involved, our study integrates Mrp2 expression and activity with concurrent assessments of microbiota composition, endotoxemia, inflammation, and OS. This strategy allows us to determine whether dysbiosis acts as an upstream regulatory factor in Mrp2 impairment—an aspect not previously examined and essential for identifying the initial drivers of its dysregulation, as well as the mediating factors involved. The absence of studies linking transporter function with microbiota-dependent inflammatory and redox pathways highlights a clear gap in the current literature and underscores the need for the present investigation, providing crucial insights into elucidating the molecular basis of Mrp2 dysregulation and identifying potential therapeutic targets in obesity.

Collectively, the findings of the present study reveal a mechanistic association between IM and Mrp2 function, connecting xenobiotic clearance with the regulation of intestinal immune and redox systems, and emphasizing the role of Mrp2 in contributing to the maintenance of intestinal functional integrity.

## 2. Materials and Methods

### 2.1. Chemicals

1-chloro-2,4-dinitrobenzene (CDNB), glutathione, fluorescein isothiocyanate-dextran (FD-4), 2-vinylpyridine, thiobarbituric acid, nitroblue tetrazolium (NTB), pepstatin A, phenylmethylsulfonyl fluoride and hydrogen peroxide were purchased from Sigma-Aldrich (St. Louis, MO, USA). Inulin (dahlia tubers-derived, ≥90% purity) was obtained from Santa Cruz Biotechnology Inc. (Dallas, TX, USA). 2-Vinylpyridine was obtained from Fluka Chemical Corp (Milwaukee, WI, USA). All other chemicals and reagents used were commercial products of analytical-grade purity.

### 2.2. Animals and Treatments

Male C57BL/6 mice (5 weeks old, 20–25 g) were obtained from the Centro de Investigación y Producción de Reactivos Biológicos (CIPReB), School of Medicine, National University of Rosario, Santa Fe, Argentina.

Initially, animals were randomly assigned to two groups: control (C) and high-fat diet (HFD). The C group was fed a standard chow diet purchased from GEPSA^®^ (http://www.gepsa.com), with the following centesimal composition: protein (24%), fat (6%), fiber (7%), calcium (1–1.2%), phosphorus (0.5–0.9%), minerals (8%), and moisture (13%). To induce obesity, mice in the HFD group were fed an HFD containing 40% more fat (*w*/*w*) for 8 weeks, prepared according to previously described formulations [36,37,38]. The fat source used to formulate the HFD was Faty^®^ beef fat (Quickfood S.A., Buenos Aires, Argentina), which is primarily composed of beef-derived fat and includes antioxidants such as BHA (INS 320), BHT (INS 321), and citric acid (INS 330) for preservation. Its fatty acid profile consists of approximately 51 g/100 g saturated fats, 37 g/100 g monounsaturated fats, 6 g/100 g polyunsaturated fats, and 6 g/100 g trans fats.

**Figure 1:**
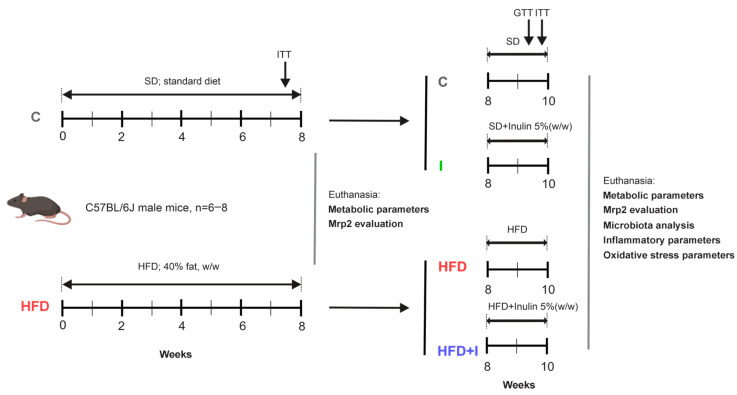
**Schematic representation of treatment protocol.** The duration of the protocol was 10 weeks in total. The initial 8 weeks correspond to the installation of the obese model, which was verified by the insulin tolerance test (ITT), 3 days before the end of the treatments, and metabolic parameters (physiological and biochemical parameters): C and HFD groups. Treatment with inulin was initiated at the end of week 8 and continued for 2 weeks, while the HFD was maintained until the end: C, HFD, HFD+I, and I groups. Glucose tolerance (GTT) and insulin tolerance (ITT) tests were performed 5 and 3 days before the end of the treatments, respectively.

After the first 8-week phase, once obesity was confirmed in HFD group based on body weight (BW) gain and metabolic assessments, animals were randomly reassigned to two groups (*n* = 6–8 per group): HFD group and HFD plus inulin (HFD+I). Animals that received standard chow diet, were randomly reassigned to two groups (*n* = 6–8 per group): C (control group) and I (inulin alone treatment). The C group continued receiving the standard diet throughout the entire experimental period. The HFD group remained on the HFD for a total of 10 weeks. The HFD+I group received the HFD during the initial 8 weeks (obesity induction phase), followed by 2 additional weeks on the same diet supplemented with 5% (*w*/*w*) inulin. The I group consumed the standard diet for 8 weeks and then the same diet supplemented with 5% (*w*/*w*) inulin for the final 2 weeks. A schematic overview of these two consecutive treatment phases is shown in Figure 1.

Mice were housed six per cage under controlled conditions (23 ± 2 °C; 12 h light/dark cycle) with free access to tap water and food. BW (grams) and calorie intake (kilocalories per animal) were measured.

Five and three days before the end of the treatments, glucose tolerance (GTT) and/or insulin tolerance (ITT) tests were performed, respectively, to evaluate glucose metabolism and confirm the development of insulin resistance [21,37].

All the experimental protocols were performed according to the Regulation for the Care and Use of Laboratory Animals and were approved by the Institutional Animal Use Committee of the National University of Rosario, Argentina (CUDI N° 26233/2023, Resolution 651/2023).

### 2.3. Specimen Collection

At the end of the treatments, animals were weighed and were fasted for 12 h before euthanasia, which was performed under intraperitoneal (i.p.) anesthesia (ketamine, 200 mg/kg BW; midazolam, 5 mg/kg BW) between 08:00 and 09:00 h. After laparotomy, blood samples were collected by cardiac puncture into heparinized tubes to measure plasma glucose, triacylglycerol, total cholesterol, and endotoxin levels (Section 2.4 and Section 2.11). Epididymal, abdominal, and retroperitoneal fats were excised and weighed; values were expressed as a percentage of final BW. The entire small intestine was removed from the pyloric sphincter to the ileocecal valve. The first 4 cm starting from the pyloric valve, corresponding to the duodenum, were excluded, and the next part was considered proximal jejunum [36]. This segment was gently flushed with ice-cold saline and dried using filter paper. For Western blot studies, the jejunum was immediately opened lengthwise, the mucus layer was carefully removed, and the mucosa was scraped, weighed, and used for brush border membrane (BBM) preparation (Section 2.5) [21]. For confocal microscopy analysis of Mrp2 localization, small intestinal segments were frozen in liquid 1,1,1,2-tetrafluoroethane (Electroquímica DELTA SRL, Buenos Aires, Argentina) and used for slice preparation (Section 2.6) [39]. For total RNA isolation, small sections from the jejunum were cut, snap-frozen in liquid nitrogen, and kept at −70 °C until further processing (Section 2.7). For Mrp2 in vitro transport studies, segments of the jejunum were used fresh to prepare everted intestinal sacs (Section 2.8) [21]. Fecal samples were collected and immediately frozen at −70 °C for gut microbiota analysis (Section 2.9) [40]. In addition, non-everted segments were used for permeability assays and ROS detection (Section 2.10 and Section 2.12) [39]. Proximal jejunum aliquots were homogenized in ice-cold saline (1:2) for assessment of lipid peroxidation and antioxidant enzyme activities (Section 2.12 and Section 2.13) [21].

### 2.4. Biochemical Assays

Plasma glucose, triglyceride, and cholesterol levels were determined spectrophotometrically using enzymatic kits (Wiener Lab, Rosario, Argentina) according to the manufacturer’s instructions, with a Jasco V-730 spectrophotometer (JASCO Corporation, Hachioji, Tokio, Japón) [35]. All biochemical measurements were performed in duplicate, and internal quality controls provided by the manufacturer were used to minimize technical variability and ensure measurement accuracy.

The glucose tolerance test (GTT) was performed 5 days before sacrifice. A glucose bolus (2 g/kg BW in saline solution, i.p.) was administered to conscious animals after a 12 h fasting. Glycemia was measured in blood collected from the tail prior to glucose injection (time 0) and at 30-, 60-, 90-, and 120-min post-injection using enzymatic kits (Wiener Lab, Rosario, Argentina), with absorbance determined spectrophotometrically. The area under the curve (AUC) was calculated using GraphPad Prism 8, and results were expressed as mM·min [21].

For the insulin tolerance test (ITT), performed 3 days before the experiments, mice were fasted for 6 h before the experiment, and a blood sample was collected from the tail vein to determine basal glycemia (0 min). The animals were then injected i.p. with 0.75 U/kg human recombinant insulin (Denver Farma S.A., Buenos Aires, Argentina), and additional blood samples were collected at 30, 60, and 90 min to determine glycemia using enzymatic kits (Wiener Lab, Rosario, Argentina), with absorbance determined spectrophotometrically. The area under the curve (AUC) was calculated using GraphPad Prism 8, and results were expressed as mM·min [21].

### 2.5. Western Blot Analysis

The BBMs were prepared from mucosal scrapings. Protein concentration was measured using bovine serum albumin as a standard [41]. Aliquots of the BBM preparations were kept on ice and used the same day for Western blot analysis. Mrp2 was detected in BBMs as described previously [1]. Equal amounts of protein (20 µg) were separated by SDS–PAGE and transferred to PVDF membranes. Uniformity of loading was verified by Ponceau S staining and β-actin detection. Blots were probed with anti-MRP2 (M2 III-6, Alexis Laboratories, San Diego, CA, USA), anti-occludin (71–1500, ZYMED), and anti-β-actin (A-2228, Sigma-Aldrich, St. Louis, MO, USA), followed by appropriate HRP-conjugated secondary antibodies. Immunoreactive bands were detected using a chemiluminescence kit (Pierce™ ECL Western blotting Substrate, Thermo Scientific, Rockford, IL, USA), and quantification was performed using ImageJ version win64 software.

### 2.6. Microscopy Studies

For in situ immunodetection of Mrp2, intestinal rings from jejunum were sectioned (thickness, 5 μm) and fixed as described previously [21,39]. Mrp2 was detected with the respective antibody, and the cell nuclei were detected with 4,6-diamidino-2-phenylindole (DAPI) as described previously [21,39]. All confocal studies were performed with a Nikon C1 Plus microscope (Nikon Corporation, Tokyo, Japan). To ensure comparable staining and image capture performance for the different groups belonging to the same experimental protocol, intestinal slices were prepared on the same day and mounted on the same glass slide.

### 2.7. Real-Time Polymerase Chain Reaction (PCR) Studies

Mrp2, IL-1β, and IL-6 gene expression were measured in the jejunum by real-time PCR, and results were normalized to the 36B4 gene. Total RNA was extracted from the jejunum using TRIzol reagent (Invitrogen, Carlsbad, CA, USA) according to the manufacturer’s instructions. Reverse transcription was performed with 5 µg of RNA using the Omniscript RT kit (Qi-agen, Venlo, The Netherlands). Real-time PCR was carried out using Power SYBR Green Master Mix (Solis BioDyne, Tartu, Estonia) on a StepOnePlus system (Applied Biosystems, Thermo Fisher Scientific Inc., San Jose, CA, USA). The primer sequences were as follows: Abcc2 forward 5′-accttccacgtagtgatcct-3′, reverse 5′-acctgctaagatggacggtc-3′; IL-1β forward 5′-ttgacggaccccaaaagatg-3′, reverse 5′-agaaggtgctcatgtcctca-3′; IL-6 forward 5′-cttccatccagttgccttcttg-3′, reverse 5′-tgggagtggaatcctctgtgaagt-3′; and 36B4 (RPLP0, ribosomal protein lateral stalk subunit P0) forward 5′-gtaacccgttgaaccccatt-3′, reverse 5′-ccatccaatcggtagtagcg-3′. Relative mRNA expression was determined using the 2^−ΔΔCT^ method [21].

### 2.8. Assessment of Mrp2 Activity In Vitro

Mrp2 transport activity was assessed using the everted intestinal sac model. The jejunal segments (3 cm) were everted and incubated for 30 min in the presence of 200 μM CDNB in the mucosal compartment as described previously [42]. After diffusion of CDNB into the enterocyte, and further endogenous conjugation with glutathione, the accumulation of the product dinitrophenyl-S-glutathione (DNP-SG) in the mucosal compartment was quantified by high-performance liquid chromatography.

### 2.9. Intestinal Microbiome Analysis

Fecal samples were collected before euthanasia and stored at −80 °C. DNA extraction was performed using the QIAamp DNA Stool Mini Kit (Qiagen, Hilden, Germany), followed by 16S rRNA gene sequencing (V3–V4) using the Illumina MiSeq platform. Microbial composition was analyzed using QIIME2 [43,44]. Sequencing reads were processed using QIIME2 for quality and adapter filtering, following the standardized pipeline described by Bolyen et al. [45]. Taxonomic classification was performed with Kraken2 using the SILVA reference database, and species-level abundance estimates were refined with Bracken, following the workflow described by Lu et al. [46]. Diversity analyses were conducted in R: α-diversity indices (Chao1, Shannon) and β-diversity (PCoA based on Bray–Curtis dissimilarity) were calculated using the phyloseq package [47] (Figure S8). Count normalization prior to multivariate analyses was performed using the DESeq2 package [48]. Visualizations were generated using microViz [49] and ggplot2 [50].

### 2.10. Assessment of Intestinal Paracellular Permeability

Paracellular permeability was studied using the FD-4 probe (4 kDa) permeation rate through the small intestine as described [44]. Non-everted intestinal sacs (3 cm) were filled with 1 mg/mL FD-4 and incubated for 30 min at 37 °C. The fluorescence intensity of FD-4 samples in the same mucosal compartment was determined by excitation at 485 nm and emission at 535 nm using a DTX 880 multimode detector plate reader (Beckman Coulter, Brea, CA, USA) [39].

### 2.11. Assessment of Plasma Endotoxin Levels

Plasma endotoxin levels were determined using a limulus amebocyte lysate (LAL) kit according to the manufacturer’s instructions (Lonza, Walkersville, MD, USA). Absorbance was measured at 405 nm using a microplate reader (Beckman Coulter DTX 880 Multimode Detector, Brea, CA, USA), and endotoxin concentrations were calculated from a standard curve generated with known concentrations of *Escherichia coli* O111:B4 endotoxin.

### 2.12. Assessment of Lipid Peroxidation and ROS Production

Lipoperoxidation (LPO) was assessed by measuring TBARS in intestinal homogenates as described previously [21,39,51]. ROS production was quantified using the fluorescent probe 2′,7′-dichlorodihydrofluorescein diacetate (DCFH-DA). Briefly, intestinal segments (3 cm) were filled with 5 µM DCFH-DA and incubated for 30 min at 37 °C with 5% CO_2_. Subsequently, the sacs were washed, and the mucosa layer was scraped. The 2′,7′-dichlorofluorescein (DCF) formation in supernatant was monitored at an excitation wavelength of 488 nm and an emission wavelength of 525 nm using a DTX 880 multimode detector plate reader [52].

### 2.13. Assessment of Antioxidant Enzyme Activities

Superoxide dismutase (SOD) activity was determined spectrophotometrically from the inhibition of NBT reduction, while catalase (CAT) activity was measured by monitoring the decomposition rate of H_2_O_2_ at 240 nm, as previously described [39].

### 2.14. Statistical Analysis

Data are expressed as mean ± standard deviation, and as % of control group. Statistical comparisons were made by one-way ANOVA followed by the Tukey post hoc test, except for the 8-week treatment studies, which were analyzed using Student’s *t*-test. *p* < 0.05 was considered statistically significant. All analyses were conducted using GraphPad Prism 3.1 (GraphPad Software, San Diego, CA, USA).

Pearson correlation analysis was used to evaluate the relationship between the IM and metabolic, inflammatory, OS, paracellular barrier and Mrp2 parameters.

## 3. Results

### 3.1. Establishment of the HFD-Induced Obesity Model

**Figure 2:**
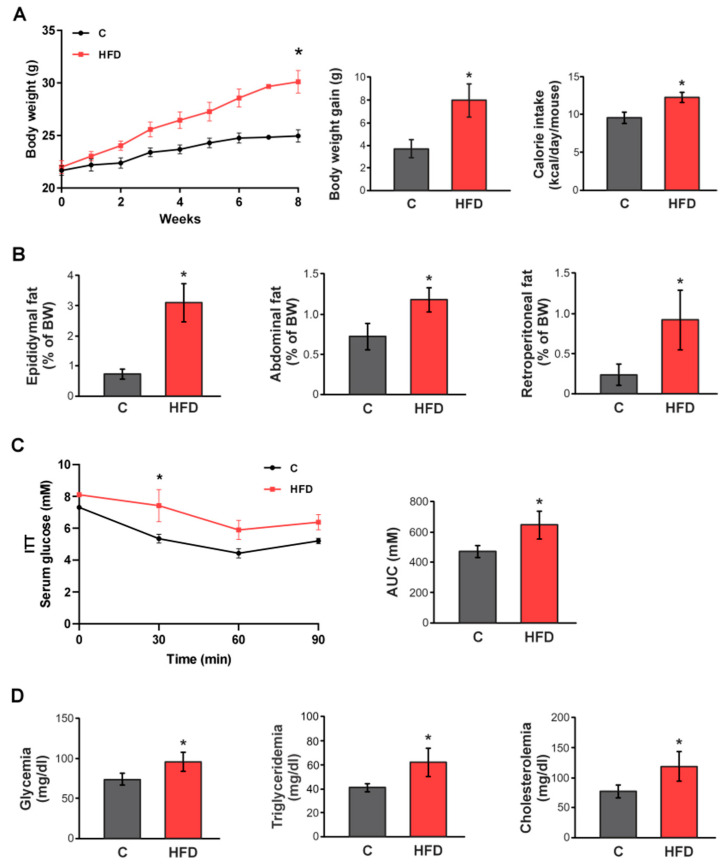
**Establishment of the HFD-induced obesity model.** (**A**) Temporal evolution of body weight (BW), cumulative weight gain (g), and caloric intake (kcal/day/mouse) in male C57BL/6 mice during 8 weeks of treatments. (**B**) Amount of epididymal, abdominal, or retroperitoneal fat was calculated as fat weight × 100/final BW. (**C**) Response curves during the intraperitoneal insulin tolerance (ITT) tests. Bar graph depicts quantification of cumulative glucose clearance in the ITT by integration of the AUC in 90 min. (**D**) Glycemia, triglyceridemia, and cholesterolemia after 8 weeks of treatments. Results are expressed as mean ± standard deviation (*n* = 6–8). * Significantly different from C group, *p* < 0.05.

To verify the establishment of the obese model, we evaluated BW and energy intake in control and HFD-fed mice. Animals subjected to the HFD displayed a significant increase in BW gain (+115%) compared to C group. Regarding caloric consumption, HFD-fed mice showed a marked increase (+28%) relative to control animals (Figure 2A). Furthermore, the HFD regimen resulted in greater adiposity, as evidenced by significantly higher weights of epididymal, abdominal, and retroperitoneal fat pads (+312%, +62%, and +270%, respectively) (Figure 2B).

In agreement with previous reports on diet-induced obesity models [53,54,55], feeding male C57BL/6 mice a HFD for 8 weeks led to elevated blood glucose levels and impaired insulin sensitivity, as demonstrated by significantly higher ITT AUC values (Figure 2C). Plasma triglyceride and cholesterol levels were also significantly increased in HFD-fed animals (Figure 2D). Overall, these findings confirmed that the HFD regimen successfully established a diet-induced obesity model in mice.

### 3.2. Effect of HFD on Mrp2 Expression and Activity

**Figure 3:**
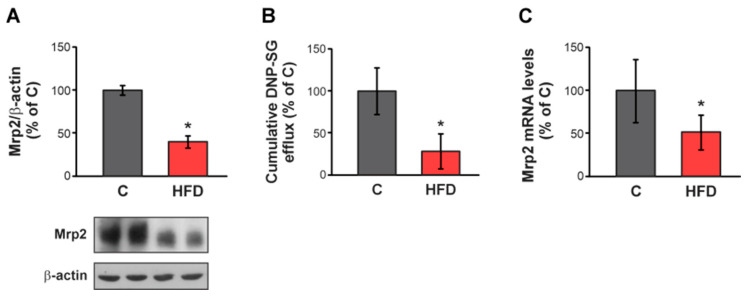
**Effect of HFD on Mrp2 expression and activity.** (**A**) Western blot analysis of Mrp2 in BBM from jejunum. Equal amounts of total protein (20 μg) were loaded in all lanes. Uniformity of protein loading and transfer from gel to PVDF membrane was controlled with Ponceau S and detection of β-actin. Densitometry data were related to β-actin. (**B**) Cumulative DNP-SG content in the mucosal compartment of everted intestinal sacs after 30 min of incubation with CDNB. (**C**) RT-qPCR assessment of Mrp2 mRNA levels in jejunum. The results were normalized to the 36B4 mRNA as the housekeeping gene. Data are presented as % of controls (C) and expressed as mean ± standard deviation (*n* = 6–8). * Significantly different from C, *p* < 0.05.

A significant decrease in Mrp2 protein expression was observed in HFD-fed mice (−60%) compared to C group (Figure 3A). Consistently, the efflux of the Mrp2 model substrate DNP-SG, generated from its precursor CDNB, was reduced (−70%) in HFD animals relative to the control group (Figure 3B). Additionally, RT-qPCR studies were conducted to assess the involvement of a transcriptional mechanism, which revealed that HFD administration reduced Mrp2 mRNA levels compared to C group (−48%) (Figure 3C). These results indicated that HFD-induced obesity led to a marked downregulation of Mrp2 expression and activity, at least in part at the transcriptional level, suggesting a compromised intestinal efflux function in this model.

### 3.3. Effect of HFD and Inulin Treatment on the Intestinal Microbiota

**Figure 4:**
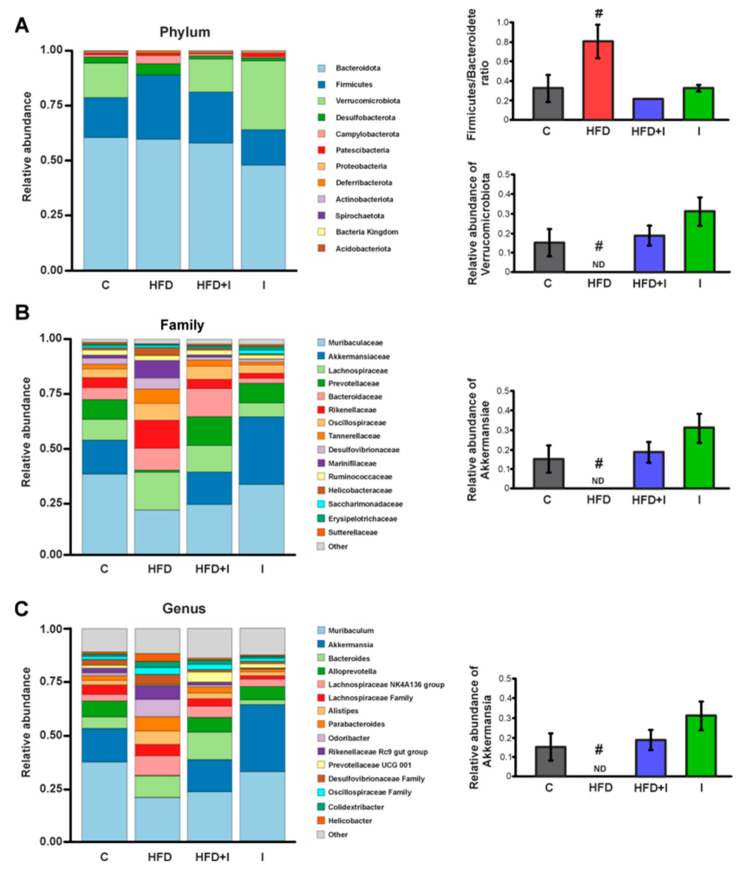
**Effect of high fat diet feeding and Inulin treatment on intestinal microbiota.** Relative abundance of cecal microbiota from male C57BL/6 mice: (**A**) at phylum level and Firmicutes/Bacteroidetes ratio; (**B**) at family level; and (**C**) at genus level. Data are expressed as mean ± standard deviation (*n* = 3–4). #, significantly different from C, HFD+I and I groups, *p* < 0.05. ND = non-detected.

As shown in Figure 4A, Firmicutes and Bacteroidetes were the predominant phyla within the IM of all animals. Notably, the Firmicutes/Bacteroidetes ratio was markedly increased in HFD-fed mice (+141%) and was restored to baseline levels in fecal content following inulin treatment. In addition, the abundance of Verrucomicrobiota in the fecal content of HFD-fed mice was depleted. Consistently, Akkermansiaceae, a representative family of the Verrucomicrobiota phylum, was completely absent in the HFD group compared with the other groups (Figure 4B). At the genus level, the HFD-induced Akkermansia depletion was restored by inulin treatment in the HFD+I group (Figure 4C).

### 3.4. Effect of HFD and Inulin Treatment on Physiological Parameters and Energy Intake

**Figure 5:**
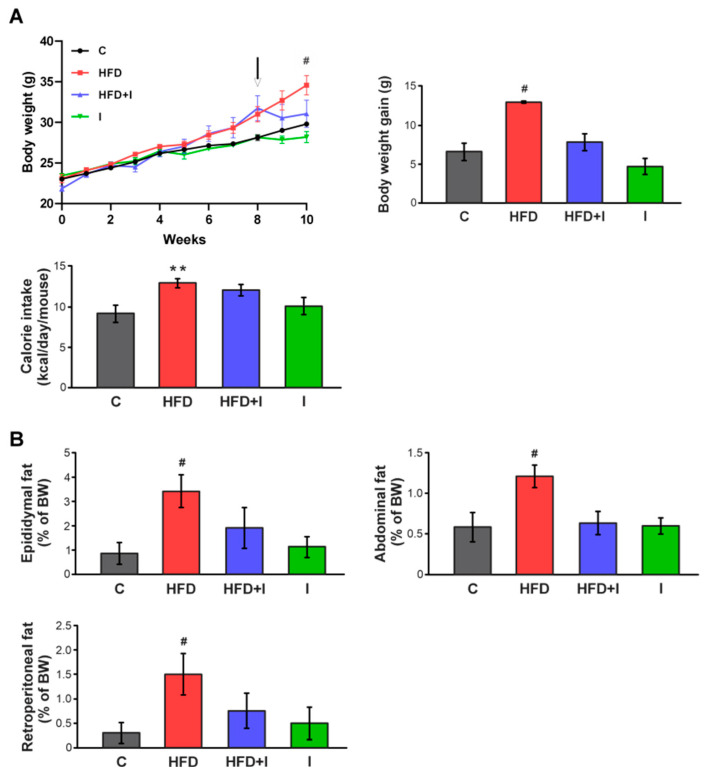
**Effect of HFD and Inulin treatment on physiological parameters and energy intake.** (**A**) Temporal evolution of body weight (BW) cumulative weight gain (g) and calorie intake (kcal/day/mouse) in male C57BL/6 mice during 10 weeks of treatments. The arrow indicates the beginning of Inulin supplementation; (**B**) Amount of epididymal, abdominal, or retroperitoneal fat was calculated as fat weight × 100/final BW. Data are presented as % of controls (C) and expressed as mean ± standard deviation (*n* = 6–8). # Significantly different from C, HFD+I and I groups, *p* < 0.05. ** Significantly different from C and I groups, *p* < 0.05.

After 10 weeks of treatment, the increase in BW gain observed in HFD-fed mice (+93%) was attenuated by inulin supplementation during the last two weeks of treatment (Figure 5A). As expected, calorie intake was significantly higher in the HFD group (+40%) compared with controls, while co-treatment with the prebiotic partially reduced this value (Figure 5A). Fat consumption also led to greater adiposity compared to C group, as evidenced by a significant increase in epididymal, abdominal, and retroperitoneal fat weights (+290%, +105%, and +380%, respectively) (Figure 5B). Conversely, HFD-fed mice supplemented with inulin exhibited a marked reduction in fat accumulation, approaching levels observed in the control group. These findings indicate that inulin supplementation effectively mitigates HFD-induced weight gain and adiposity, suggesting a beneficial role in regulating energy balance.

### 3.5. Effect of HFD and Inulin Treatment on Biochemical Parameters

**Figure 6:**
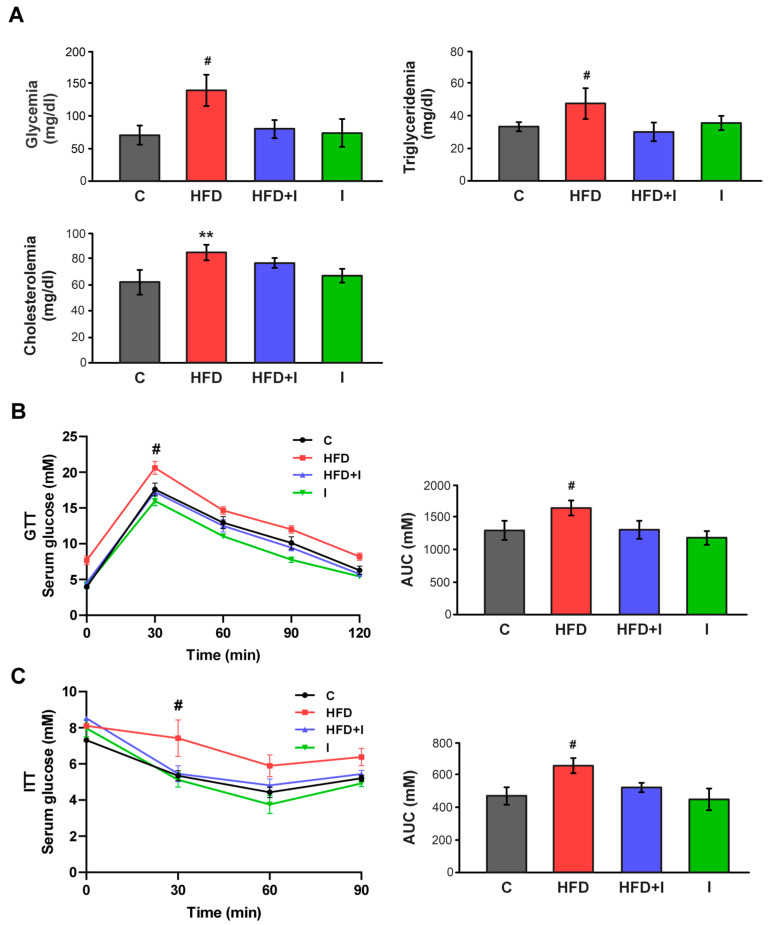
**Effect of HFD and Inulin treatment on biochemical parameters.** (**A**) Glycemia, triglyceridemia, and cholesterolemia after 10 weeks of treatments; (**B**,**C**) Response curves during the intraperitoneal glucose and insulin tolerance tests (GTT and ITT, respectively). Bar graphs depict quantification of cumulative glucose clearance in the GTT and ITT by integration of the AUC in mM/120 min and mM/90 min, respectively. Results are expressed as mean ± standard deviation (*n* = 6–8). ** Significantly different from C and I groups, *p* < 0.05. # Significantly different from C, HFD+I and I groups, *p* < 0.05.

Figure 6A shows that, after 10 weeks of treatment, plasma glucose and triglyceride levels were increased in HFD mice (+94% and +43%, respectively), while they were completely normalized by inulin treatment. Regarding plasma total cholesterol levels, no significant differences were observed between the HFD and HFD+I groups (Figure 6 A). Once again, the HFD group exhibited higher glycemia levels in both the GTT and ITT curves, as indicated by significantly elevated AUC values (+26% and +37%, respectively) compared with controls. Inulin treatment significantly improved the response to glucose overload and exogenous insulin administration in HFD-fed mice, suggesting a reversal of insulin resistance (Figure 6B and Figure 6C, respectively). Consistent with the normalization of physiological parameters described above, these findings indicated that inulin supplementation effectively reversed HFD-induced metabolic alterations.

### 3.6. Effect of HFD and Inulin Treatment on Intestinal Paracellular Barrier and Plasma Endotoxin Levels

**Figure 7:**
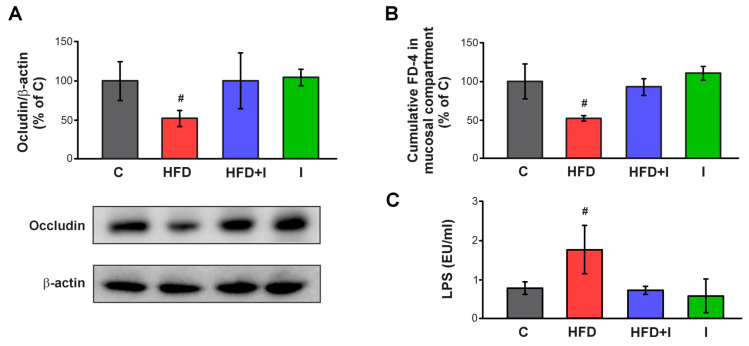
**Effect of HFD and Inulin treatment on intestinal paracellular barrier and plasma endotoxin levels.** (**A**) Western blot analysis of occludin in the BBM fraction. Equal amounts of total protein (20 µg) were loaded in gels. Uniformity of protein loading and transfer from gel to PVDF membranes were controlled with Ponceau S and detection of β-actin. Densitometry data was related to β-actin; (**B**) paracellular permeation of FD-4 across intestinal barrier. FD-4 accumulated in the mucosal compartment was measured after 30 min of intestinal sacs incubation; (**C**) plasma endotoxin levels were determined using a limulus amebocyte lysate (LAL) kit). Results are expressed as mean ± standard deviation (*n* = 6–8). # Significantly different from C, HFD+I and I groups, *p* < 0.05.

Occludin expression, a key tight junction protein involved in regulating the paracellular route, was also evaluated. Figure 7A shows that HFD treatment markedly reduced occludin expression (−48%) compared with controls, whereas inulin supplementation restored it to control levels. Furthermore, intestinal paracellular permeability was assessed in non-everted intestinal segments using the non-permeable macromolecule FD-4. The accumulation of this fluorescent probe in the mucosal compartment was significantly reduced (−48%) in HFD-fed animals, indicating increased paracellular permeability, while co-treatment with inulin normalized FD-4 flux (Figure 7B). As expected, plasma LPS levels were markedly elevated (+127%) in HFD-fed mice compared with controls, reflecting a state of metabolic endotoxemia. Remarkably, inulin supplementation during the final two weeks of treatment completely reversed this effect, restoring plasma LPS levels to baseline (Figure 7C). These findings demonstrated that inulin supplementation preserved intestinal paracellular integrity and prevented HFD-induced endotoxin translocation, aligning with the restoration of physiological and metabolic parameters and supporting a systemic protective role of inulin via paracellular barrier maintenance.

### 3.7. Effect of HFD and Inulin Treatment on Intestinal Proinflammatory Cytokine Levels

**Figure 8:**
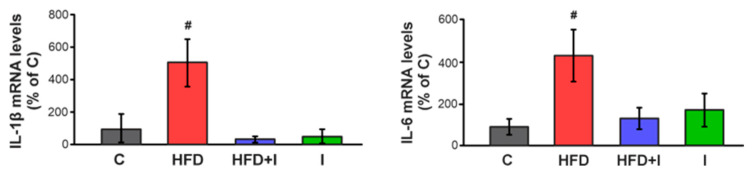
**Effect of HFD and Inulin treatment on intestinal proinflammatory cytokine levels.** RT-qPCR assessment of IL-1β (left) and IL-6 (right) mRNA levels in jejunum homogenates. The results were normalized to the 36B4 mRNA as the housekeeping gene. Data are presented as % of controls (C) and expressed as mean ± standard deviation (*n* = 6–8). # Significantly different from C, HFD+I and I groups, *p* < 0.05.

Figure 8 shows that the levels of both IL-1β and IL-6 were significantly elevated in the proximal jejunum of the HFD group compared with the C group (+413% and +335%, respectively). Co-treatment with inulin completely normalized the levels of both cytokines. These findings demonstrated that inulin supplementation effectively reversed the HFD–induced inflammatory state in intestinal tissue by reestablishing pro-inflammatory cytokine levels.

### 3.8. Effect of HFD and Inulin Treatment on Intestinal Redox Balance and Antioxidant Defenses

**Figure 9:**
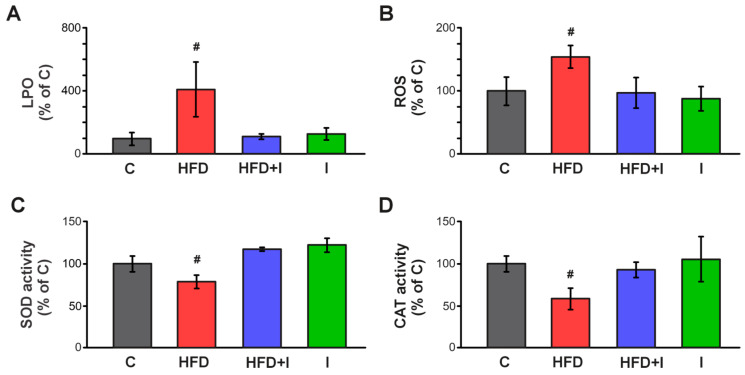
**Effect of HFD and Inulin treatment on intestinal redox balance and antioxidant defenses.** Lipoperoxidation (LPO), quantified by thiobarbituric acid reactive substances (TBARS) (**A**), reactive oxygen species (ROS) (**B**), superoxide dismutase (SOD) (**C**) and catalase (CAT) activity (**D**) were determined in jejunum homogenates. Data are presented as % of controls (C) and expressed as mean ± standard deviation (*n* = 6–8). # Significantly different from C, HFD+I and I groups, *p* < 0.05.

Figure 9A shows that the HFD-induced increase in TBARS levels (+330%) returned to normal values after inulin administration. Intracellular ROS production was significantly higher in obese mice (+54%), whereas it was completely normalized in the HFD+I group (Figure 9B). Regarding antioxidant defenses, the HFD-induced decrease in SOD activity (−21%) was fully restored with inulin supplementation (Figure 9C). Similarly, CAT activity was reduced (−41%) in obese mice, and inulin co-treatment significantly improved this OS marker compared with controls (Figure 9D). These results demonstrated that inulin supplementation effectively restored intestinal redox balance and antioxidant defenses, preventing HFD-induced OS, which is commonly associated with the inflammatory process.

### 3.9. Effect of HFD Administration and Inulin Treatment on Intestinal Mrp2

**Figure 10:**
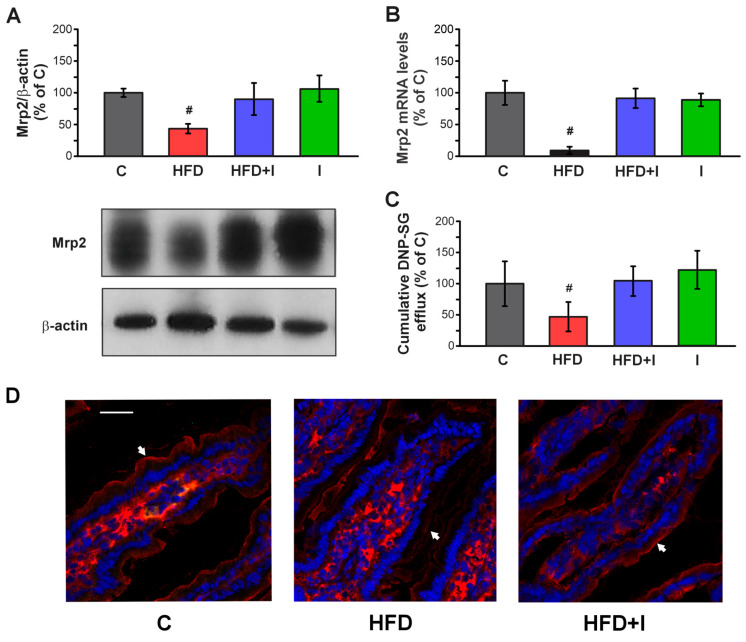
**Effect of HFD administration and Inulin treatment on intestinal Mrp2.** (**A**) Western blot analysis of Mrp2 in the BBM fraction. Equal amounts of total protein (20 ug) were loaded in gels. Uniformity of protein loading and transfer from gel to PVDF membranes were controlled with Ponceau S and detection of β-actin. Densitometry data was related to β-actin. (**B**) RT-qPCR analysis of Mrp2 mRNA levels. The results were normalized to the 36B4 mRNA as the housekeeping gene. (**C**) Cumulative DNP-SG content in the mucosal compartment of everted intestinal sacs after 30 min of incubation with CDNB. (**D**) Confocal microscopy detection of Mrp2 in the intestinal villus. Mrp2 was labeled with red fluorescence (white arrow) and nuclei were stained with DAPI Scale bar is indicated in white and correspond to 50 μm. Data are presented as % of controls (C) and expressed as mean ± standard deviation (*n* = 6). #, significantly different from C, HFD+I and I, *p* < 0.05.

HFD administration for 10 weeks caused a significant decrease in Mrp2 protein expression (−57%) in the proximal jejunum compared to C group, as previously observed after 8 weeks of HFD exposure in this study. Importantly, this reduction was reversed in mice co-treated with inulin (Figure 10A). Additionally, analysis of mRNA levels in the obese mice revealed a decrease in Mrp2 expression compared with controls (−90%), whereas this parameter returned to normal levels in the HFD+I group (Figure 10B). Similarly, the cumulative DNP-SG content in the mucosal compartment was reduced by 53% in the HFD group and was restored under co-treatment conditions. Inulin alone did not affect Mrp2 expression or activity compared with control mice (Figure 10C). Lastly, Mrp2 expression in the villus was analyzed in situ by confocal immunofluorescence microscopy (Figure 10D). In the HFD group, Mrp2 showed diffuse localization rather than the well-defined apical distribution observed in control mice, and its immunoreactivity signal was weaker. This alteration was reversed by inulin.

These results demonstrated that inulin supplementation effectively restored Mrp2 expression, localization, and function in HFD-fed mice, potentially contributed to the normalization of intestinal detoxification capacity.

### 3.10. Correlation Analysis Between Akkermansia Abundance and Metabolic, Inflammatory, OS, Paracellular Barrier and Mrp2 Parameters

**Figure 11:**
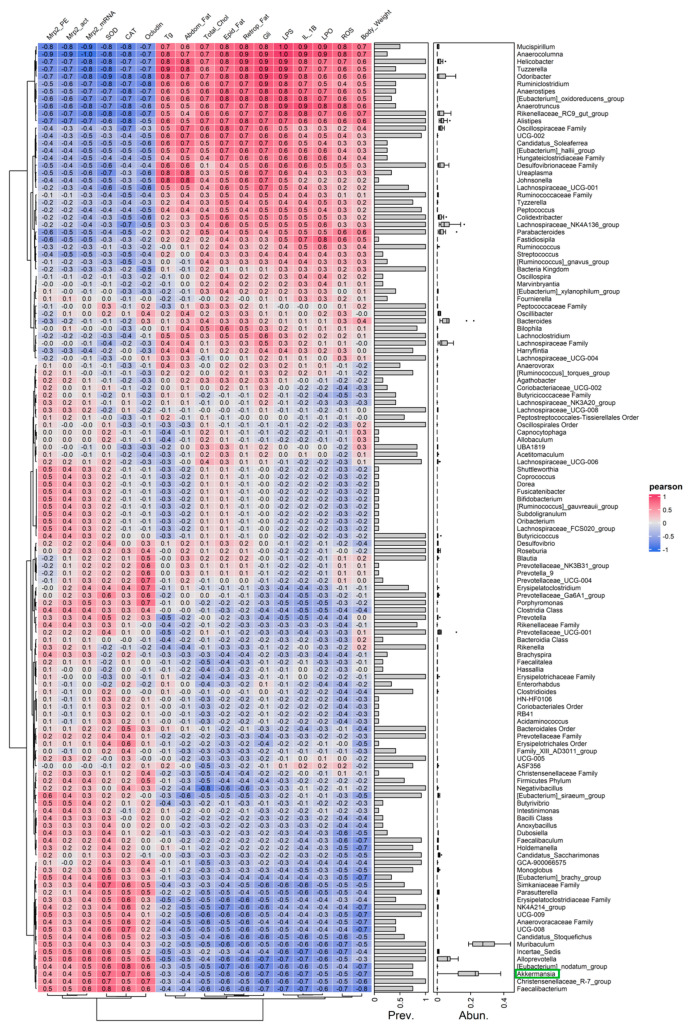
**Correlation analysis between IM composition and metabolic, inflammatory, oxidative stress, paracellular barrier and Mrp2 parameters.** Pearson’s correlation heatmap showing associations between the relative abundance of intestinal bacterial taxa and host metabolic, inflammatory, OS, paracellular barrier, and Mrp2 parameters in male C57BL/6 mice. Color scale indicates correlation coefficients (blue: negative; red: positive). Akkermansia is highlighted with a green box. These results showed that the abundance of Akkermansia was positively correlated Mrp2 mRNA levels, MRP2 protein expression and activity, SOD and CAT activities, and occludin expression, while being negatively correlated with IL-1β, LPO, and ROS levels. These findings indicate that greater Akkermansia abundance (such as observed in the HFD+I group in Figure 4) could be associated with reduced inflammation and OS, an improved metabolic profile, and enhanced intestinal paracellular integrity and Mrp2-dependent biochemical barrier function.

To further examine the findings described above, we performed Pearson correlation analyses to evaluate the relationships between IM composition, focusing on Akkermansia that is distinguishably distributed across treatment groups, and markers of metabolism, inflammation, OS, paracellular barrier integrity, and intestinal Mrp2. This analysis is presented in Figure 11, where the color scale from blue to red represents increasing correlation coefficients.

## 4. Discussion

In the present study, we addressed the interrelationship between HFD-induced obesity, intestinal dysbiosis, and intestinal Mrp2 regulation using the prebiotic inulin. This well-established beneficial modulator of the IM was employed as an experimental tool to assess the IM contribution to Mrp2 regulation. To achieve this, we employed a murine model, as human and rodent IM are comparable at the phylum level, and HFD feeding reproduces key features of obesity, allowing the assessment of its consequences [56,57]. Given that Mrp2 plays a crucial role in maintaining the intestinal biochemical barrier and thus protecting the organism from potentially harmful xenobiotics and endobiotics, it is essential to understand how obesity-induced intestinal alterations affect this transport system. In addition, considering that long-term oral therapies are often required to manage obesity-associated comorbidities [24,58], alterations in this transporter could potentially have important implications for drug absorption, disposition and efficacy.

Initial experiments conducted to validate the obesity model revealed that mice subjected to a HFD for 8 weeks developed marked hyperglycemia, hypertriglyceridemia, hypercholesterolemia, increased adiposity, excessive BW gain, and insulin resistance. In this context, the elevation in blood glucose is not a reflection a higher sugar content in the HFD, as carbohydrate levels are comparable to those of the standard diet. The elevated blood glucose levels observed in HFD animals denote the onset of peripheral insulin resistance, a well-established metabolic consequence of high-fat feeding that limits glucose uptake and leads to fasting hyperglycemia [59,60]. Taken together, these alterations confirm the establishment of a diet-induced obesity model under the present experimental protocol.

Significantly, under these conditions, Mrp2 expression in the jejunum, its main site of expression and the primary site of nutrient absorption, was markedly reduced at both protein and mRNA levels, suggesting that its regulation may occur, at least in part, at transcriptional level. More critically, this downregulation resulted in a substantial loss of transport activity, as demonstrated by the decreased apical excretion of its model substrate, DNP-SG. This impairment of the Mrp2-dependent biochemical barrier may have important consequences by leading to the tissue accumulation of toxic endogenous metabolites and xenobiotics, as well as by potentially affecting the bioavailability and safety of clinically relevant substrate drugs, under obesity-related pathological conditions.

Based on the available evidence, these findings are particularly relevant, as this constitutes the first comprehensive study characterizing the alteration of intestinal Mrp2 in the context of diet-induced obesity, assessing Mrp2 expression (mRNA and protein) as well as functional activity. To date, only the studies conducted by Lu et al. (2019) [35] and our previous work [37] have investigated this topic, each employing different HFD-induced obesity models (24 and 16 weeks of HFD feeding, respectively). Both studies reported a downregulation of intestinal Mrp2 mRNA expression; however, they remain limited in scope. Lu et al. (2019) [35] did not evaluate functional consequences or the underlying molecular mechanisms involved. Furthermore, and importantly, no studies to date have specifically examined the potential key regulatory mechanisms responsible for intestinal Mrp2 modulation in obesity, still less whether intestinal dysbiosis represents a key initiating factor in this regulatory process. The absence of studies linking Mrp2 function with microbiota-dependent inflammatory and redox pathways highlights a clear gap in the current literature and underscores the necessity of the present investigation to elucidate primary factors contributing to its dysregulation.

As previously described, HFD-induced intestinal dysbiosis involves an increase in LPS-producing bacteria accompanied by a reduction in beneficial genera associated with preserving barrier function [61]. This imbalance is associated with alterations in the SCFA profile, metabolites essential for maintaining intestinal homeostasis. Together, these alterations initiate early intestinal disturbances, including increased pro-inflammatory cytokines secretion, associated OS, disruption of epithelial integrity, and enhanced permeability. These events facilitate the translocation of LPS into the systemic circulation, leading to metabolic endotoxemia and, consequently, systemic inflammation and OS, which ultimately contribute to the metabolic dysfunctions characteristic of diet-induced obesity, as demonstrated in our model. Importantly, endotoxemia, OS, and pro-inflammatory cytokines are recognized regulators of intestinal drug transporter expression and activity [19,39,52,62]. Crucially, recent evidence has demonstrated that intestinal microbiota–driven inflammatory and oxidative pathways can modulate ABC efflux transporters, including Mrp2, primarily through regulatory mechanisms dependent on FXR and Nrf2, as described in models of metabolic dysfunction-associated steatohepatitis and toxin-induced liver injury [29,30,31]. These findings provide a solid theoretical foundation and support the mechanistic plausibility of the proposed microbiota–inflammation–oxidative stress axis for the intestinal Mrp2 regulation, as the restoration of microbial balance in these models has been observed to reduce inflammation and oxidative stress while concomitantly normalizing the expression and function of Mrp2 and other ABC transporters. Nevertheless, whether these factors contribute to Mrp2 downregulation in the context of obesity-associated dysbiosis remains to be elucidated.

Interestingly, as previously reported [63], inulin supplementation can mitigate HFD-induced intestinal dysbiosis, improving intestinal barrier function and reducing inflammation, at least in part, through SCFA modulation. These improvements in intestinal homeostasis are associated with the attenuation of obesity-associated systemic metabolic dysfunctions. In this context, we propose that this dietary intervention, by initially correcting intestinal dysbiosis and thus interrupting the subsequent cascade of pathological events, may not only mitigate alterations in Mrp2 but also implicate the IM as a central regulatory component, acting through downstream mediators such as inflammation and OS in the mechanisms underlying its modulation. Accordingly, we conducted a sequential evaluation of the metabolic and intestinal alterations in HFD-fed mice, in comparison with mice receiving inulin supplementation.

Based on the 16S rRNA sequencing results and consistent with the well-documented microbial signature of obesity, characterized by an increased Firmicutes-to-Bacteroidetes ratio, the HFD group displayed a significant rise in Firmicutes and a concomitant decrease in Bacteroidetes, along with a marked reduction in Verrucomicrobiota, and the complete depletion of the genus *Akkermansia*. Inulin supplementation effectively counteracted these alterations, normalizing the Firmicutes/Bacteroidetes ratio and restoring *Akkermansia* abundance, as previously reported [43]. These changes in intestinal microbiota paralleled substantial improvements in systemic metabolic parameters: inulin attenuated BW gain, reduced adiposity, normalized plasma glucose and triglyceride levels, and significantly improved glucose and insulin tolerance, while plasma cholesterol remained unaffected. Altogether, these findings support previous evidence indicating that inulin supplementation beneficially remodels the IM and ameliorates obesity-associated metabolic disturbances [43,64,65].

In turn, under our experimental conditions, intestinal dysbiosis led to increased intestinal permeability, as demonstrated by a significant rise in paracellular FD-4 permeation in the HFD group, an effect that was at least partially attributable to lower expression of the tight junction protein, occludin. Consistent with these findings, endotoxin plasma levels were significantly higher in HFD-fed mice, confirming elevated circulating LPS and supporting an increased proportion of LPS-containing microbiota in the intestine, as demonstrated [14]. Following the sequence of events, we evaluated both the local induction of an inflammatory response and the associated pro-oxidant status in the intestinal tissue of HFD-fed mice. This analysis revealed a marked up-regulation of the pro-inflammatory cytokines IL-1β and IL-6. Moreover, this inflammatory process was accompanied by a redox imbalance, as evidenced by increased levels of LPO and ROS, and reduced activities of the antioxidant enzymes SOD and CAT in the HFD group. Importantly, inulin administration effectively reversed the increase in intestinal permeability, restored occludin expression, and reduced the elevation in circulating LPS levels, while concomitantly attenuating both inflammation and OS in HFD-fed mice. Altogether, these correlations support an evident interconnection between IM, endotoxemia, and local inflammation and OS in intestinal tissue during HFD feeding and further indicate that positive modulation of the MI by administration of the inulin prebiotic can effectively counteract these downstream pathophysiological alterations. This is consistent with previous observations in visceral adipose tissue from obese mouse models, where modulation of the MI normalized LPS levels and mitigated inflammatory and OS markers [15]. This is also in agreement with prior studies demonstrating the beneficial effects of inulin on the specific parameters evaluated in the present work [43,64,65].

More significantly, the observed intestinal alterations occurred concomitantly with the downregulation of Mrp2 at both protein and transcriptional levels, resulting in a notable reduction in efflux activity. Moreover, our data suggest that the IM may play a central role in modulating this transporter, acting as an initiating factor within the regulatory network. The previously observed normalization of the IM after inulin treatment further supports this hypothesis, as it coincided with the reversal of the Mrp2 alterations. Additional support for this interpretation is substantiated by Zhou et al. (2022) [32], who reported that in antibiotic-treated rats, perturbations of the IM alters the pharmacokinetics of cyclosporine A, at least in part by down-regulating intestinal P-gp and Mrp2 expressionu. This effect was reversed following fecal microbiota transplantation, highlighting the interconnection between microbial composition and the regulation of intestinal ABC transporters. Even though their experimental model differs from ours, and their results are limited by the lack of specification of the intestinal region analyzed and the absence of an evaluation of the specific impact on Mrp2 activity using a model substrate, these findings are particularly valuable. They underscore the relevance of our study’s original contribution by providing the first functional characterization of Mrp2 impairment and its direct correlation with the sequential progression from initial dysbiosis to subsequent inflammatory and oxidative events in the context of diet-induced obesity.

In turn, although we provide key and novel information derived from our experimental approach using inulin, we recognize that direct evidence validating dysbiosis as the central link in the negative regulation of Mrp2 is still lacking. Nevertheless, two additional observations provide further strong support for this possibility: (i) we previously demonstrated that jejunal Mrp2 expression and activity are markedly reduced in a FRD-induced MS model in rats, where inflammation and OS emerged as critical drivers of this impairment [20,21], and further identified OS as a key mediator of the Mrp2 post-translational regulation [39,52]; and (ii) Di Luccia et al. [22] showed that in FRD-fed rats, the development of MS was directly correlated with IM alterations, as treatment with antibiotics or fecal samples significantly reduced metabolic, inflammatory, and OS markers. Together, these evidence suggest that altered IM may contribute to the regulation of Mrp2 not only under FRD conditions but also in the present HFD model.

On another note, although few studies have demonstrated that bacteria play a direct role in the regulation of ABC transporters, they have been found to exhibit significant correlations with several factors involved in the regulation of these proteins. For instance, *Clostridium_sensu_stricto_1* showed positive correlations with the inflammatory cytokines IL-6 and TNF-α [66]. More importantly, in our study, Pearson’s correlation analysis revealed that the abundance of Akkermansia, known for its anti-inflammatory properties [67], exhibited a consistent association pattern with key inflammatory and OS parameters and with intestinal Mrp2. Specifically, Akkermansia showed negative correlations with pro-inflammatory cytokines and OS markers, and positive correlation with intestinal Mrp2, further supporting the role of OS and inflammation as key mediating factors. Moreover, previous studies have shown that Akkermansia can upregulate the production of SCFAs, which in turn modulate the expression of ABC transporters [68]. These relationships are consistent with the emerging concept that microbial metabolites, including SCFAs and bile acid-derived molecules, modulate efflux transporters through FXR- and Nrf2-mediated pathways [29,30,31], reinforcing the mechanistic concept that inflammation and OS act as central mediators linking intestinal dysbiosis to Mrp2 downregulation under HFD conditions. Therefore, future qualitative and quantitative analyses of microbial-derived metabolites will be essential to further elucidate the underlying mechanism in greater detail.

Finally, some limitations should be acknowledged. The 10-week duration of the protocol may not fully reflect the long-term effects of HFD or sustained inulin supplementation, as chronic exposure to HFD is known to progressively alter gut microbiota composition, inflammation, and intestinal barrier function [14,69]. In addition, only male mice were included. Sex-dependent differences in the IM, immune responses, and the regulation of ABC transporters have been consistently reported [70,71,72]. Therefore, future studies with longer exposure periods and the inclusion of both sexes would help better determine the robustness and generalizability of these findings.

## 5. Conclusions

In conclusion, our findings provide the first evidence identifying intestinal dysbiosis, inflammation, and OS as a central regulatory axis of intestinal Mrp2 in obesity, highlighting the IM as a key modulator. Unlike previous studies conducted in the context of obesity, our study integrates Mrp2 expression, transport activity, microbiota composition, intestinal permeability, endotoxemia, inflammation, and OS. This comprehensive approach constitutes an important innovation, enabling the identification of dysbiosis as a potential key determinant of Mrp2 impairment, an aspect not previously explored and essential for elucidating the early regulatory events underlying its dysfunction. Future studies are needed to fully elucidate the precise underlying molecular mechanisms, including their relative contributions and causal hierarchy. Extending these findings, the reversal of dysbiosis and intestinal alterations by inulin underscores its potential as a therapeutic strategy, not only for metabolic disorders but also for restoring the Mrp2-dependent intestinal biochemical barrier. Given the essential role of Mrp2 in this barrier, its dysfunction under dysbiotic conditions may have pivotal physiological implications by facilitating the accumulation of endogenous and exogenous toxic compounds, and, because of its broad range of drug substrates, could also influence their pharmacokinetics and therapeutic efficacy.

## Data Availability

The original contributions presented in this study are included in the article and the Supplementary Materials. Further inquiries can be directed to the corresponding author.

## Supplementary Materials

The following supporting information can be downloaded at: https://www.mdpi.com/article/10.3390/pharmaceutics17121575/s1, Figure S1: Mrp2 expression by Western blot, corresponding to Figure 3; Figure S2: β-actin expression by Western blot, corresponding to Figure 3; Figure S3: Ocludin expression by Western blot, corresponding to Figure 7; Figure S4: β-actina expression by Western blot, corresponding to Figure 7; Figure S5: Mrp2 expression by Western blot, corresponding to Figure 10; Figure S6: β-actin expression by Western blot, corresponding to Figure 10; Figure S7: Confocal microscopy detection of Mrp2, corresponding to Figure 10. Figure S8: Rarefaction_diversity; Information S1: Results summary of microbiota studies.

## Abbreviations

The following abbreviations are used in this manuscript:

ABC	ATP-Binding Cassette
AUC	Area Under the Curve
BBM	Brush Border Membranes
BW	Body weight
C	Control
CAT	Catalase
CDNB	1-chloro-2,4-dinitrobenzene
DCF	2′,7′-dichlorofluorescein
DCFH-DA	Fluorescent probe 2′,7′-dichlorodihydrofluorescein diacetate
DNP-SG	dinitrophenyl-S-glutathione
FD-4	fluorescein isothiocyanate-dextran
FRD	Fructose-Rich Diet
GTT	Glucose Tolerance Test
HFD	High-Fat Diet
I	Inulin
IM	Intestinal Microbiota
ITT	Intraperitoneal Insulin Tolerance Test
LPO	Lipoperoxidation
LPS	Lipopolysaccharide
Mrp2/Abcc2	Multidrug resistance-associated protein 2
MS	Metabolic Syndrome
NBT	Nitroblue tetrazolium
OS	Oxidative Stress
ROS	Reactive Oxygen Species
SCFAs	Short-Chain Fatty Acids
SOD	Superoxide dismutase
TBARS	Thiobarbituric Acid Reactive Substances
*w*/*w*	Weight to weight
