# Supplementary material for: Inulin Reverses Intestinal Mrp2 Downregulation in a Diet-Induced Obesity Mouse Model: Role of Intestinal Microbiota as a Pivotal Modulator
Source: Pharmaceutics. 2025 Dec 6;17(12):1575. doi: 10.3390/pharmaceutics17121575 (PMC12736295; doi:10.3390/pharmaceutics17121575)
Supplement: Supplementary file 1 [file pharmaceutics-17-01575-s001.zip › Figure_S8_Rarefaction_diversity.pdf]

Figure S8: Rarefaction diversity data analysis

0.1 Rarefaction curves

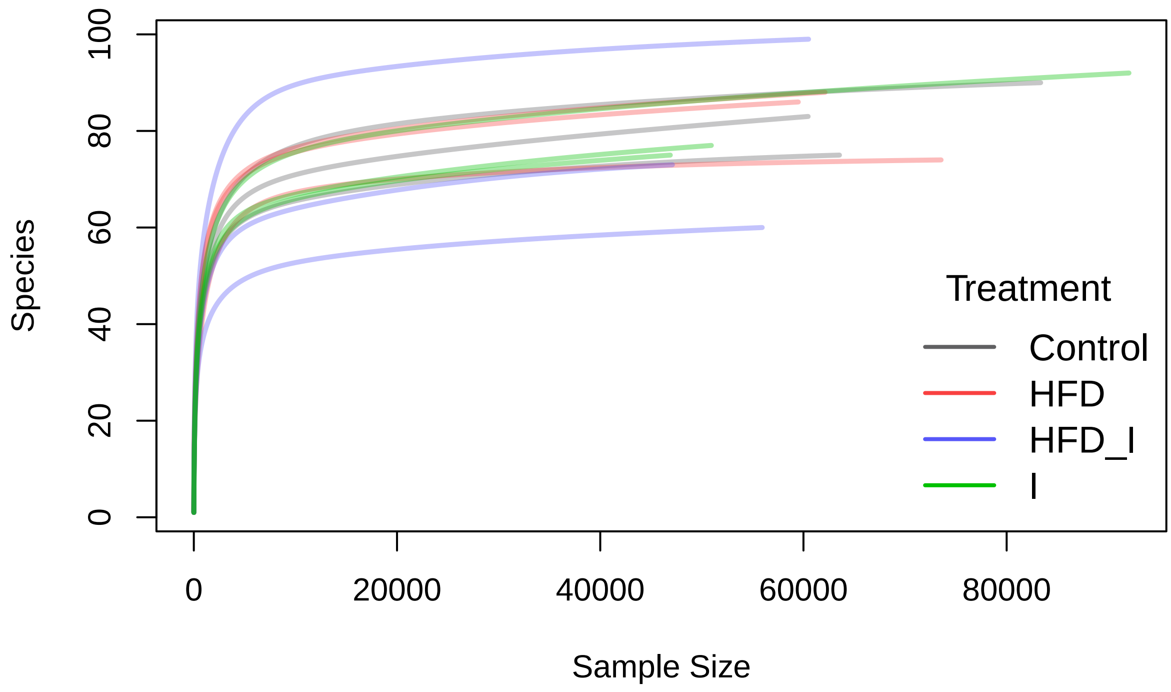

Lower sample size 47542.

0.2 α-Diversity

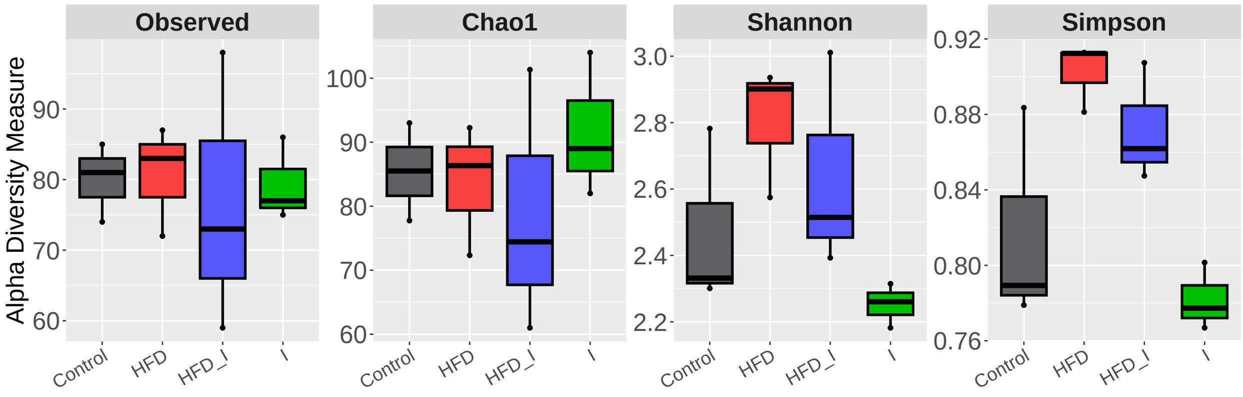

The Kruskall-Wallis test paired with a dunn post-hoc test was apllied to determine significant differences.

0.2.1 Observed

```
##        Comparison          Z    P.unadj P.adj
```

```
## 1   Control - HFD -0.1132277 0.9098500     1
## 2 Control - HFD_I  0.4529108 0.6506130     1
## 3     HFD - HFD_I  0.5661385 0.5712996     1
## 4     Control - I -0.1132277 0.9098500     1
## 5         HFD - I  0.0000000 1.0000000     1
## 6       HFD_I - I -0.5661385 0.5712996     1
```

0.2.2 Chao 1

```
##        Comparison          Z   P.unadj     P.adj
## 1   Control - HFD  0.2264554 0.8208472 0.8208472
## 2 Control - HFD_I  0.5661385 0.5712996 0.8569494
## 3     HFD - HFD_I  0.3396831 0.7340952 0.8809142
## 4     Control - I -0.5661385 0.5712996 1.0000000
## 5         HFD - I -0.7925939 0.4280144 1.0000000
## 6       HFD_I - I -1.1322770 0.2575180 1.0000000
```

0.2.3 Shannon

```
##        Comparison          Z    P.unadj      P.adj
## 1   Control - HFD -1.3587324 0.17423139 0.34846278
## 2 Control - HFD_I -0.9058216 0.36503027 0.43803633
## 3     HFD - HFD_I  0.4529108 0.65061296 0.65061296
## 4     Control - I  1.1322770 0.25751798 0.38627697
## 5         HFD - I  2.4910095 0.01273807 0.07642843
## 6       HFD_I - I  2.0380987 0.04154007 0.12462020
```

0.2.4 Simpson

```
##        Comparison          Z     P.unadj      P.adj
## 1   Control - HFD -1.6984156 0.089429359 0.26828808
## 2 Control - HFD_I -0.7925939 0.428014450 0.42801445
## 3     HFD - HFD_I  0.9058216 0.365030272 0.54754541
## 4     Control - I  0.9058216 0.365030272 0.43803633
## 5         HFD - I  2.6042372 0.009207901 0.05524741
## 6       HFD_I - I  1.6984156 0.089429359 0.17885872
```

None of the observed differences resulted significant.

0.3 β - Diversity

We visualize beta diversity by making a PCoA (Principal Components Analysis) ordination.

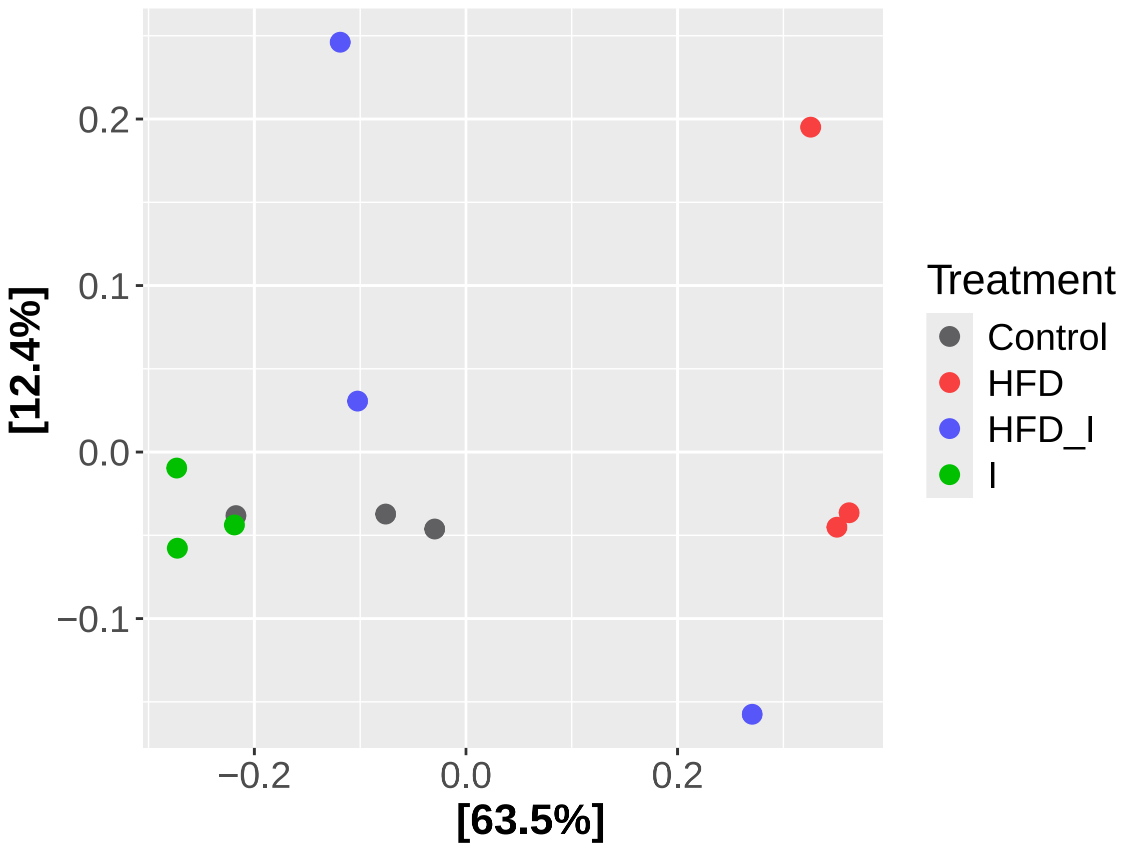

A certain degree of separation is observed between groups, particularly between the **HFD** group and the others, suggesting differences in microbial community composition.

0.3.1 Overall Permanova

```
## Permutation test for adonis under reduced model
## Permutation: free
## Number of permutations: 999
##
## adonis2(formula = bray_dist ~ Treatment, data = sam, permutations = 999)
##          Df SumOfSqs      R2      F Pr(>F)
## Model     3  0.72623 0.65078 4.9694  0.001 ***
## Residual  8  0.38971 0.34922
## Total    11  1.11593 1.00000
## ---
## Signif. codes:  0 '***' 0.001 '**' 0.01 '*' 0.05 '.' 0.1 ' ' 1
```

0.3.1.1 PERMANOVA Results Interpretation Beta-diversity analysis based on Bray–Curtis dissimilarities revealed a significant effect of Treatment on the overall microbial community structure (PERMANOVA, $R^2 = 0.65$, $F = 4.97$, $p = 0.002$). Approximately 65% of the variation in community composition was explained by treatment differences, indicating a clear clustering of samples according to experimental groups. Homogeneity of multivariate dispersions was assessed using betadisper, showing no significant differences among groups ($F = 3.63$, $p = 0.064$), supporting that the observed dissimilarities reflect genuine treatment effects rather than unequal within-group variability.

0.3.2 Pairwise Permanova

pairs	Df	SumsOfSqs	F.Model	R2	p.value	p.adjusted	sig
Control vs HFD	1	0.3403299	10.715966	0.7281864	0.1	0.6	
Control vs HFD_I	1	0.0882879	1.225279	0.2344907	0.4	1.0	
Control vs I	1	0.0731832	3.879819	0.4923741	0.1	0.6	
HFD vs HFD_I	1	0.2195651	2.794729	0.4113083	0.2	1.0	
HFD vs I	1	0.5557588	21.905103	0.8455903	0.1	0.6	
HFD_I vs I	1	0.1753311	2.669987	0.4002987	0.1	0.6	

No significant difference was found.

Beta-diversity analysis based on Bray–Curtis dissimilarities (after CLR transformation) revealed a significant overall effect of Treatment on gut microbiota composition (PERMANOVA, $R^2 = 0.65$, $F = 4.97$, $p = 0.002$). Pairwise comparisons did not yield significant adjusted p-values, likely reflecting limited sample size; however, large effect sizes were observed between the HFD and I groups ($R^2 = 0.85$) and between Control and HFD ($R^2 = 0.73$), indicating marked compositional shifts associated with these treatments.
