# Supplementary material for: Inulin Reverses Intestinal Mrp2 Downregulation in a Diet-Induced Obesity Mouse Model: Role of Intestinal Microbiota as a Pivotal Modulator
Source: Pharmaceutics. 2025 Dec 6;17(12):1575. doi: 10.3390/pharmaceutics17121575 (PMC12736295; doi:10.3390/pharmaceutics17121575)
Supplement: Supplementary file 1 [file pharmaceutics-17-01575-s001.zip › Supplementary_Materials-Zecchinati_2025.pdf]

Figure S1: Mrp2 expression by Western blot, corresponding to Figure 3.

Lines: 1 2 3 4 5 6 7 8 9 10 11 12

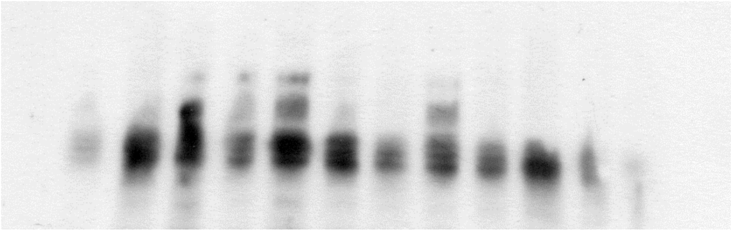

Line 1: Molecular weight marker (MWM)
Line 2: Control 1
Line 3: Control 2
Line 4: Control 3
Line 5: Control 4
Line 6: Control 5
Line 7: Control 6
Line 8: HFD
Line 9: HFD 1
Line 10: HFD 2
Line 11: HFD 3
Line 12: HFD 4

Figure S2: β-actin expression by Western blot, corresponding to Figure 3.

Lines: 1 2 3 4 5 6 7 8 9 10 11 12 13

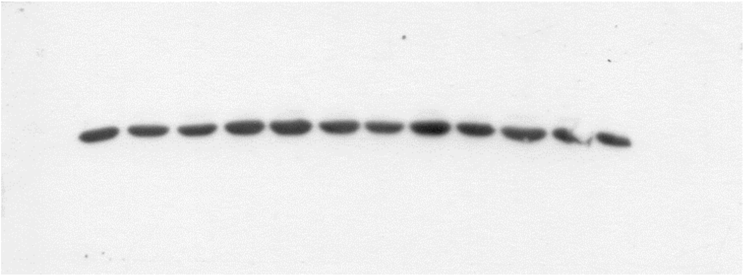

Line 1: Molecular weight marker (MWM)
Line 2: Control 1
Line 3: Control 2
Line 4: Control 3
Line 5: Control 4
Line 6: Control 5
Line 7: Control 6
Line 8: HFD
Line 9: HFD 1
Line 10: HFD 2
Line 11: HFD 3
Line 12: HFD 4

Figure S3: Ocludin expression by Western blot, corresponding to Figure 7.

Lines: 1 2 3 4 5 6 7 8 9 10 11 12 13 14 15

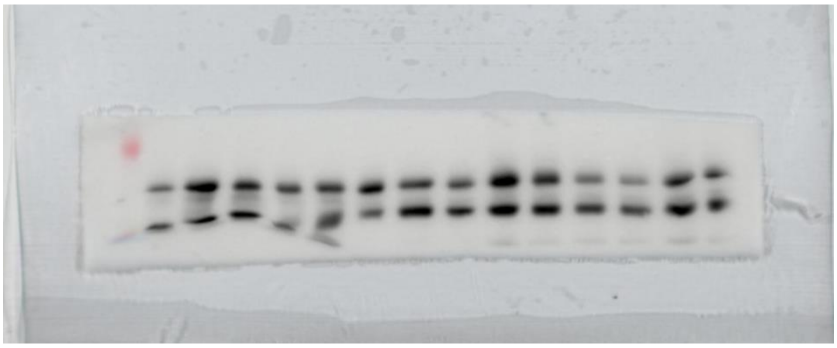

Line 1: Molecular weight marker (MWM)
Line 2: Control 1
Line 3: Control 2
Line 4: Control 3
Line 5: HFD 1
Line 6: HFD 2
Line 7: HFD 3
Line 8: HFD 4
Line 9: HFD+I 1
Line 10: HFD+I 2
Line 11: HFD 5
Line 12: HFD 6
Line 13: I1
Line 14: I2

Figure S4: β-actina expression by Western blot, corresponding to Figure 7.

Lines: 1 2 3 4 5 6 7 8 9 1(11 12 1: 14 15

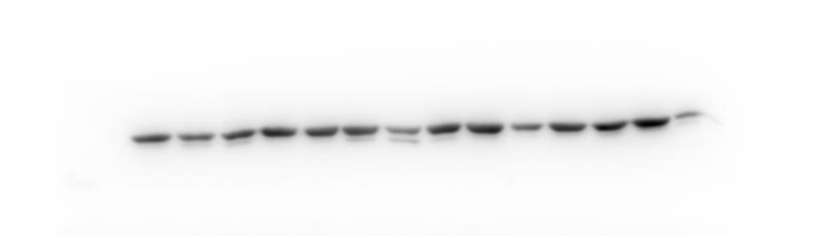

Line 1: Molecular weight marker (MWM)
Line 2: Control 1
Line 3: Control 2
Line 4: Control 3
Line 5: HFD 1
Line 6: HFD 2
Line 7: HFD 3
Line 8: HFD 4
Line 9: HFD+I 1
Line 10: HFD+I 2
Line 11: HFD 5
Line 12: HFD 6
Line 13: I1
Line 14: I2

Figure S5: Mrp2 expression by Western blot, corresponding to Figure 10.

Lines: 1 2 3 4 5 6 7 8 9 10 11

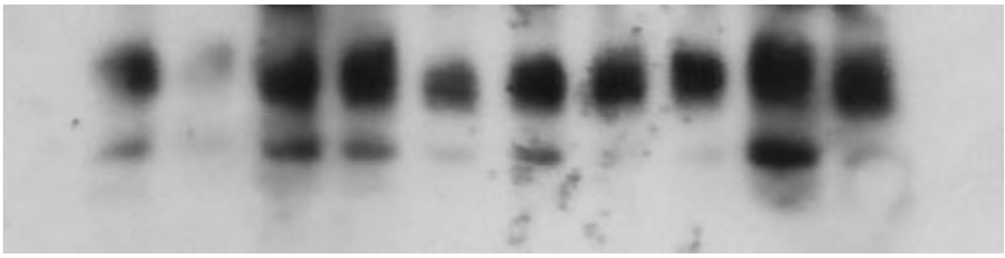

Line 1: MWM
Line 2: Control
Line 3: HFD
Line 4: HFD+I
Line 5: Control
Line 6: HFD
Line 7: HFD+I
Line 8: Control
Line 9: I
Line 10: C
Line 11: I

Figure S6: β-actin expression by Western blot, corresponding to Figure 10.

Lines: 1 2 3 4 5 6 7 8 9 10 11

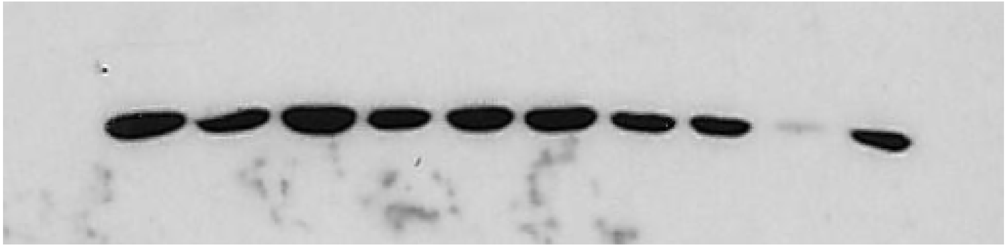

Line 1: MWM
Line 2: Control
Line 3: HFD
Line 4: HFD+I
Line 5: Control
Line 6: HFD
Line 7: HFD+I
Line 8: Control
Line 9: I
Line 10: C
Line 10: I

Figure S7: Confocal microscopy detection of Mrp2, corresponding to Figure 10.

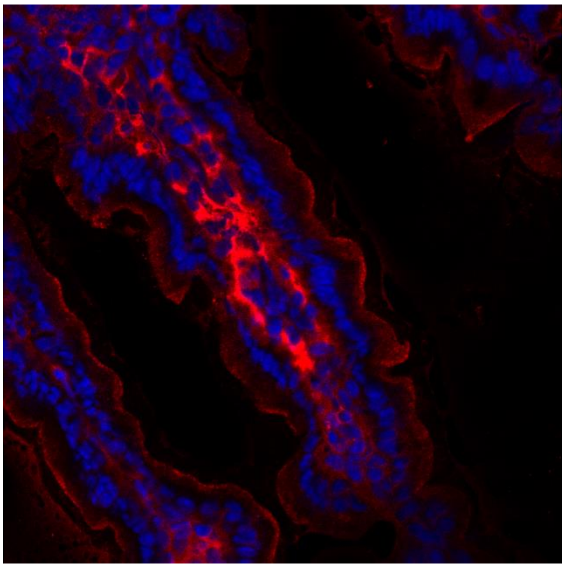

Control

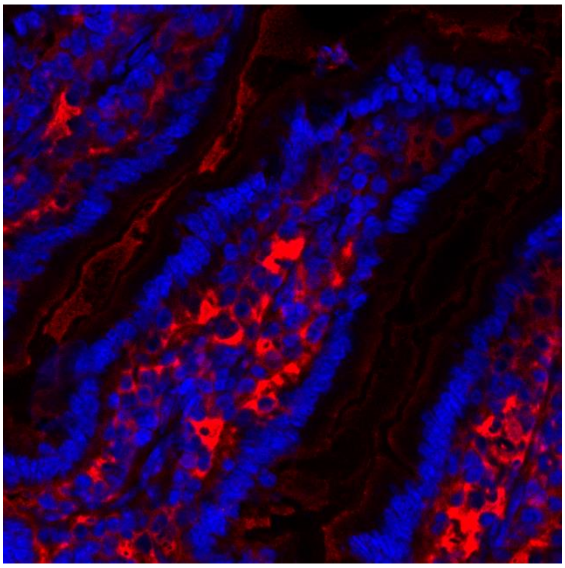

HFD

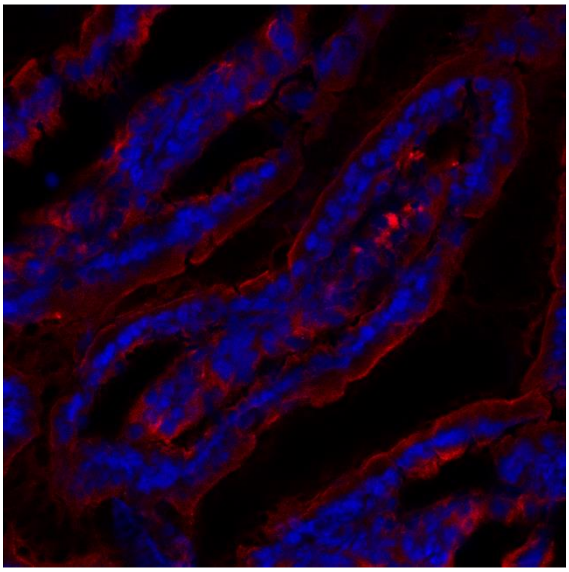

HFD+I

Information S1: Results summary of microbiota studies:

file:///C:/Users/Grupo%20ADM/Downloads/Results_summary%20(10).html#P-values
